# Analysis of the Effect of Network Structure and Disulfide Concentration on Vitrimer Properties

**DOI:** 10.3390/polym15204123

**Published:** 2023-10-17

**Authors:** Itxaso Azcune, Edurne Elorza, Alaitz Ruiz de Luzuriaga, Arrate Huegun, Alaitz Rekondo, Hans-Jürgen Grande

**Affiliations:** 1 CIDETEC, Basque Research and Technology Alliance (BRTA), Paseo Miramón 196, 20014 Donostia-San Sebastian, Spainaruiz@cidetec.es (A.R.d.L.);; 2 Advanced Polymers and Materials—Physics, Chemistry and Technology Department, University of the Basque Country (UPV/EHU), Avda. Tolosa 72, 20018 Donostia-San Sebastian, Spain

**Keywords:** vitrimers, disulfide exchange, network cross linking density, disulfide concentration

## Abstract

A set of five vitrimers with glass transition temperatures in the range of 80–90 °C were designed to assess the effect of the network structure and disulfide concentration on their dynamic and mechanical properties, and to find the best performing system overall compared to the commercial Araldite LY1564/Aradur 3486 commercial thermoset system. Vitrimer networks were prepared by incorporating mono- and bifunctional epoxy reactive diluents and an amine chain extender into the Araldite LY1564/4-aminophenyldisulfide system.

## 1. Introduction

The use of dynamic covalent bonds has emerged as a hot topic in materials science in order to obtain materials with unprecedented properties. The introduction of these dynamic covalent bonds in three-dimensional networks allows the obtainment of recyclable, reshapeable and reparable thermosets. Dynamic covalent networks can be classified in two main groups according to the exchange mechanism of the dynamic bonds present in the network: dissociative or associative networks. The first type of networks contain dissociative bonds, meaning that a bond is broken (under external stimulus) presenting a sudden viscosity drop due to a decrease in the crosslink density. This broken bond can be reformed by recovering the initial properties of the material. In associative networks the crosslink density remains constant because the bond exchange occurs in a concerted manner (the bonds are broken and reform almost at the same time) and thus, the viscosity decreases gradually with the temperature. The main difference between the two kinds of dynamic networks is that during the reshuffling of associative networks the crosslink density keeps constant while the internal stress is released due to topological rearrangement of the network. The associative networks are also called vitrimer materials and are one of the most interesting when high performance is required. Although vitrimers are crosslinked materials, their specific dynamic linkages can undergo exchange reactions resulting in materials that can be reshaped, in contrast to traditional thermosets [1,2,3,4,5]. The first reports regarding the introduction of these associative dynamic bonds in thermosets were published in 2011 [6] by Leibler, where metal-catalyzed transesterification chemistry was introduced in epoxy networks. Since then, many dynamic bonds have been introduced in thermoset materials: imine bond exchange [7], boronic esters [8], olefin metathesis [9], transamination [10] or disulfide exchange [11,12,13,14,15]. Among them, disulfide bonds are the most promising ones and are one of the most employed. The dynamic mechanism of sulfur–sulfur bonds has been extensively studied and Matxain et al. [16] demonstrated that the exchange mechanism follows a radical mediated [2 + 1] mechanism. The radical formation occurs mainly due to thermal dissociation of the disulfide bond. 

**Figure 1:**
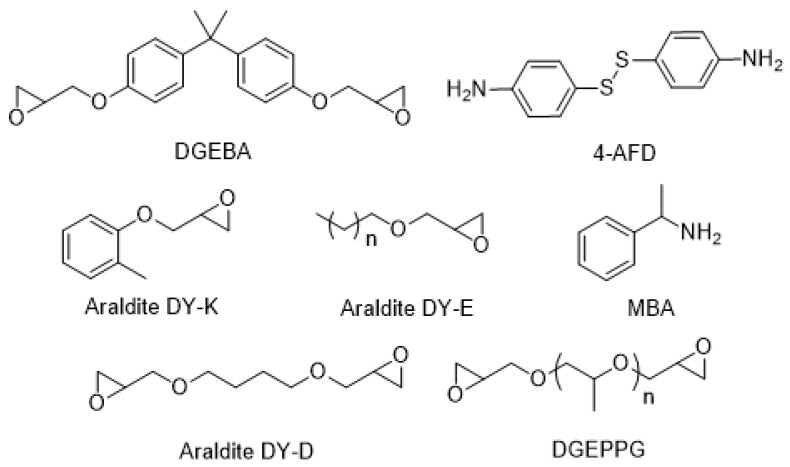
Chemical structure of the main reagents (simplification of mixtures).

The possibility of reshaping fully cured vitrimer composites opens the path to re-using end-of life parts without undertaking matrix/fiber separation processes. This recycling approach could be of special interest for glass-fiber composites for which any kind of separation of matrix and fiber will not be economically profitable. In this context, aromatic disulfide-based epoxy vitrimers have shown high potential [11,12,13,14,15]. This type of vitrimer already shows dynamic properties at 50–70 °C above the glass transition temperature (Tg). Thus, the reshaping of composites with low or moderate Tg could be achieved without risking the thermal stability of the material. A straightforward strategy to synthesize aromatic disulfide-based epoxy vitrimers is by using 4-aminophenyl disulfide (4-AFD) as a hardener and adjusting the formulation with co-reagents to match the required specifications, such as viscosity, Tg and disulfide exchange kinetics. Recently, the high tuneability and chemical control of the aromatic disulfide exchange kinetics was reported which described the effect of having a different hardener structure, free pending amine groups and a catalyst in the system [14]. The effect of Tg on the shape properties of these type of vitrimers was also reported [15]. Still, insights on the effect of the network structure, crosslink density and disulfide concentration on the dynamic and mechanical properties are required to design a vitrimer for a specific application. Here, we have addressed these important effects in order to better understand the dynamic behavior of disulfide-containing vitrimers. There are still few reports in which the influence of crosslink density has been studied [17]. In this study, we selected the thermoset Araldite LY1564 cured with Aradur 3486 as the benchmark epoxy resin. Additionally, we designed five vitrimers with matching glass transition temperatures (Tg) in the range of 80–90 °C, each exhibiting distinct network structures and varying disulfide concentrations to compare both their dynamic behavior and mechanical properties. All the vitrimer formulations were based on Huntsman Araldite LY1564-based epoxy resin and 4-AFD hardener and one of the following diluents, which differed in chemical structure and functionality: butanediol-derived bifunctional epoxy (Araldite DY-D), polypropylene glycol-derived bifunctional epoxy (DGEPPG), aromatic monofunctional epoxy (Araldite DY-K), aliphatic monofunctional epoxy (Araldite DY-E) and methylbenzylamine (MBA) (Figure 1).

The curing cycle and stoichiometric relationship between epoxy resins and hardeners have a great effect on the physical and the mechanical properties of the epoxy resins. To limit the scope of this study, the NH equivalents were set as 1.1 per epoxy group and the same curing and post-curing cycle was applied to all prepared vitrimers. 

## 2. Materials and Methods

### 2.1. Materials 

DGEBA-based epoxy resin Araldite LY1564 (epoxide equivalent weight (EEW) 161–173 g eq^−1^) and Aradur 3486 were purchased from Huntsman Advanced Materials (Basel, Switzerland). Poly(propylene glycol) diglycidyl ether (DGEPPG, Mn = 380, EEW 190 g eq^−1^) and methylbenzylamine were purchased from Sigma Aldrich (St. Louis, MO, USA). 4-Aminophenyldisulfide (4-AFD) was purchased from Carbosynth (Bratislava, Slovakia). Araldite DY-E (EEW 280–315 g eq^−1^), Araldite DY-K (EEW 167–189 g eq^−1^) and Araldite DY-D (EEW 117–125 g eq^−1^) were purchased from Ravago Chemicals (Barcelona, Spain). All chemicals were used as received.

### 2.2. Methods

Glass transition temperature (Tg) of all prepared samples was measured in a differential Scanning Calorimetry (DSC) using a TA Instruments Discovery DSC 25 Auto (TA Instruments, New Castle, DE, USA) over a temperature range from 25 °C to 190 °C under nitrogen. Tgs were obtained at a scan rate of 20 °C min^−1^ and they were calculated as the inflection point of the second heat flow step. The curing of reactive mixtures was measured at a 1 °C min^−1^ rate from ambient temperature to 200 °C; the enthalpy was measured via integration with TRIOS software (v5.6.0.87). An AR2000ex rheometer (TA Instruments, New Castle, DE, USA) from TA Instruments was used to perform the rheology characterization of the uncured formulation. Initial viscosities were measured at isothermal conditions at 10 s^−1^ for eight minutes. Thermomechanical experiments (DMA) were performed using a TA Instruments DMA Q800 equipment (TA Instruments, New Castle, DE, USA) equipped with a tensile fixture. In all cases, both the oscillation amplitude (15 μm) and frequency (1 Hz) were maintained constantly, and the samples were heated at a 3 °C min^−1^ heating rate between 25 °C and 185 °C. The temperature-dependent behavior was studied by monitoring changes in force and phase angle. Tensile stress–relaxation experiments were also performed in tensile mode. To maintain the straightness samples were initially preloaded at a force of 1 × 10^−3^ N. Samples were allowed 5 extra minutes at testing temperature to reach thermal equilibrium. After that, 1% of strain was applied to the specimens and this deformation was maintained during the test. The decrease in stress over time was recorded, and the stress relaxation modulus was calculated. Creep experiments at 60 °C were determined via a DMA Q800 instrument in tensile mode. After soaking for 2 min at 60 °C, 1 MPa constant stress was applied. The strain change was monitored over 60 min and a recovery step of 10 min was applied. Thermal stability analysis (TGA) was performed using a TA Instruments Q500 (TA Instruments, New Castle, DE, USA)equipment under air atmosphere at a heating rate of 10 °C min^−1^ from 25 °C to 800 °C. Rubber elasticity theory was applied to calculate the crosslink density (ν_C_) of all samples applying Equation (1) [v_C_ = E′/3⋅A⋅R T] where E′ is measured in the rubbery plateau (30 °C above Tg^DMA^); A is the front factor assumed to be unity; R is the gas constant (8.314 J mol^−1^ K^−1^); and T is equal to Tg^DMA^ + 30 K. Mechanical characterization of epoxy vitrimer was performed using an INSTRON 3365 Long travel Elastomeric Extensometer (Instron, Norwood, MA, USA) controlled via Bluehill Lite software (v2.21) developed by Instron (Norwood, MA, USA). Tensile tests were carried out according to the UNE-EN-ISO 527 standard [18], using dumbbell-type test specimens at an elongation rate of 2 mm min^−1^. An extensometer was used to determine tensile modulus. Flexural tests were carried out according to the UNE-EN-ISO 178 standard [19], using rectangular test specimens at 2 mm min^−1^ strain rate. An extensometer was used to determine the flexural modulus. A water absorption test was also carried out. Two specimens for each formulation where tested and before starting the water uptake tests, the specimens were dried in an oven for a 0.5 h at 120 °C, and immediately upon cooling the specimens were weighed (A). Then, specimens were immersed in water at 60 °C and when the equilibrium was reached samples were weighed (B). The water absorption is calculated as = (B − A)/A × 100%. Specimens were cut to the dimensions required for the characterization tests using a milling machine.

### 2.3. Synthetic Procedures

The weight ratio of the components of epoxy vitrimers is given in Table 1. All vitrimers were prepared according to the previously reported procedure [11]. Araldite LY1564 and a reactive diluent were heated at 60 °C and degassed under vacuum. Once the degassing was complete, the corresponding amount of hardener (4-AFD) was added under stirring at 80 °C until fully dissolved. Then, the mixture was degassed for 10 min under vacuum at 60 °C. The resulting mixture was poured into the space between the two PTFE-coated glass sheets separated with ≈1.5 mm and ≈3.5 mm rubber joints and cured at 130 °C for 1 h and post-cured at 150 °C for 1 h in a furnace. For comparison purposes, the Araldite LY1564/Aradur 3486 (weight ratio 100:34) mixture was prepared following the same synthetic procedure and cured at 80 °C for 1.5 h and at 100 °C for 5 h according to the product data sheet instructions.

## 3. Results and Discussion

### 3.1. Formulation of Aromatic Disulfide-Based Epoxy Vitrimers with Comparable Tg Values

The Araldite LY1564/Aradur 3486 was taken as the reference thermoset (E). For the preparation of vitrimers, LY1564, 4-AFD hardener and reactive diluents were mixed to adjust the final Tg value (80–90 °C). The glass transition temperature of the networks is governed by the composition and structural features such as the stiffness of the polymer segments, interchain cohesive forces and crosslinking. Therefore, to achieve networks with comparable Tg values and adjusted to the 80–90 °C range, Araldite LY1564, 4-AFD hardener and reactive diluents were mixed in the specific amounts indicated in Table 1. All vitrimers were prepared according to above explained synthetic procedure and reported by Ruiz de Luzuriaga et al. [11].

The Araldite LY1564/Aradur 3486 traditional epoxy resin was taken as the reference thermoset (E). FT-IR spectra of all prepared samples were obtained and compared (Figure S1). The peak centered between 3360 and 3340 cm^−1^ corresponds to the hydrogen-bonded OH stretching band. As can be seen in Figure S1 no significant difference in OH groups can be observed between different samples. In the case of the V2S sample a more intense band can be observed due to the higher OH groups expected in cured resin. 

Monofunctional reactive epoxy diluents reduce the crosslink density as they cap the NH groups of the hardener without contributing to the formation of the network. The plasticization effect of the long aliphatic diluent DY-E is more pronounced than the one imparted by the aromatic DY-K as evidenced by the required quantities and thus, capped the NH groups in each case. Indeed, 12 mol% and 29 mol% of the NH groups were capped in V1AL and V1AR, respectively, and thus, the crosslink density (see Table 2) was reduced by half. On the other hand, bifunctional reactive diluents contribute to the formation of the networks and present higher crosslink density than networks prepared with monofunctional epoxy diluents. The lowering of the Tg is due to the increased segment flexibility or chain length between crosslinks. When using the short and flexible DY-D diluent in V2S, a substantial replacement (68 mol%) of the LY1564 was found necessary to maintain the target Tg of around 80 °C and an increase in crosslink density was also produced (see Table 2). Vitrimer V2L, which contains a long and flexible structure on the diluent part, had 37 mol% of LY1564 replaced. [15] In VMBA vitrimer part of the dynamic hardener was replaced by a monoamine that acted as a chain extender between diepoxy segments. The addition of monoamine also reduces crosslink density. If we compare all prepared vitrimers, it can be observed that the addition of monofunctional diluents led to a decrease in crosslink density and thus, the molecular weight between crosslinks increases, generating more flexible networks. In this sense, the V2S network presents the highest crosslink density and thus the lowest molecular weight between crosslinks, creating a highly rigid network.

Along with the network structure, the effect of the disulfide concentration in each network on the dynamic and mechanical properties was studied. The disulfide concentration was calculated considering the weight percentage of 4-AFD in the formulation and the density of the cured epoxy. Three of the vitrimers (V1AL, V1AR and V2L) showed comparable disulfide concentration, whereas V2S showed the highest concentration and VMBA the lowest (see Table 2). 

In Table 3, the initial viscosities and curing data (measured by DSC) of the reactive mixtures, as well as the thermal characterization (Tg^DSC^, Tg^DMA^ and degradation temperature) of the cured vitrimers, are shown. The viscosity of uncured systems is highly dependent on the nature and amount of the reactive diluent. Thus, a range of initial viscosities were measured at various temperatures; all the formulations showed suitable viscosities to be injected at 40–60 °C (initial viscosity < 250 mPa.s). The reactivity of the reactive mixtures and curing enthalpy were measured by means of DSC at 1 °C min^−1^ rate. The formulation with a higher percentage of 4-AFD has a higher curing enthalpy (more exothermic), due to the higher number of epoxy-amine reactions taking place. The opening of oxirane rings lead to OH groups that increase the polarity of the network. This is experimentally evidenced indirectly by the water absorption of the materials (Table 3). 

**Figure 2:**
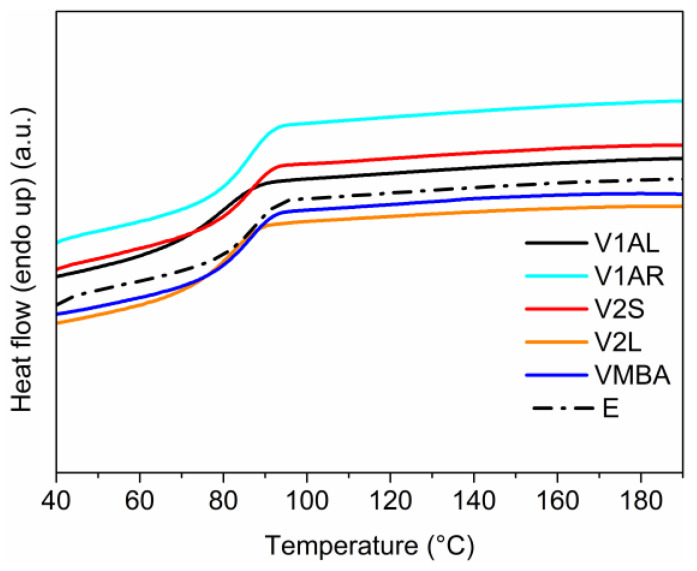
DSC thermograms.

Compared to Aradur 3486 hardener (a mixture of aliphatic and cycloaliphatic amines), the dynamic hardener (aromatic amine) shows lower reactivity and requires higher temperatures to initiate the curing reaction (thermograms in Figure S2). Indeed, the curing of vitrimers was carried out at 130 °C for 1 h and post-cured at 150 °C for 1 h. All cured vitrimers formed a homogeneous phase structure as proved by the single thermal transition (Tg) observed in DSC (Figure 2) and the complete curing of the network was confirmed by the lack of a residual exothermic signal during the first scan of the DSC thermogram. 

The thermal stability of the vitrimers was evaluated via thermogravimetric analysis under air atmosphere, and the temperature at which 5 wt.% of weight loss occurred is given in Table 3 (thermograms in Figure S3). The disulfide linkage is thermally labile compared to a C-C bond. All vitrimers decomposed in two apparent steps, and the replacement of rigid aromatic segments of LY1564 by aliphatic and flexible segments and having dangling groups decreases the overall thermal stability of the network compared to E.

### 3.2. Network Structure and Dynamic Properties

**Figure 3:**
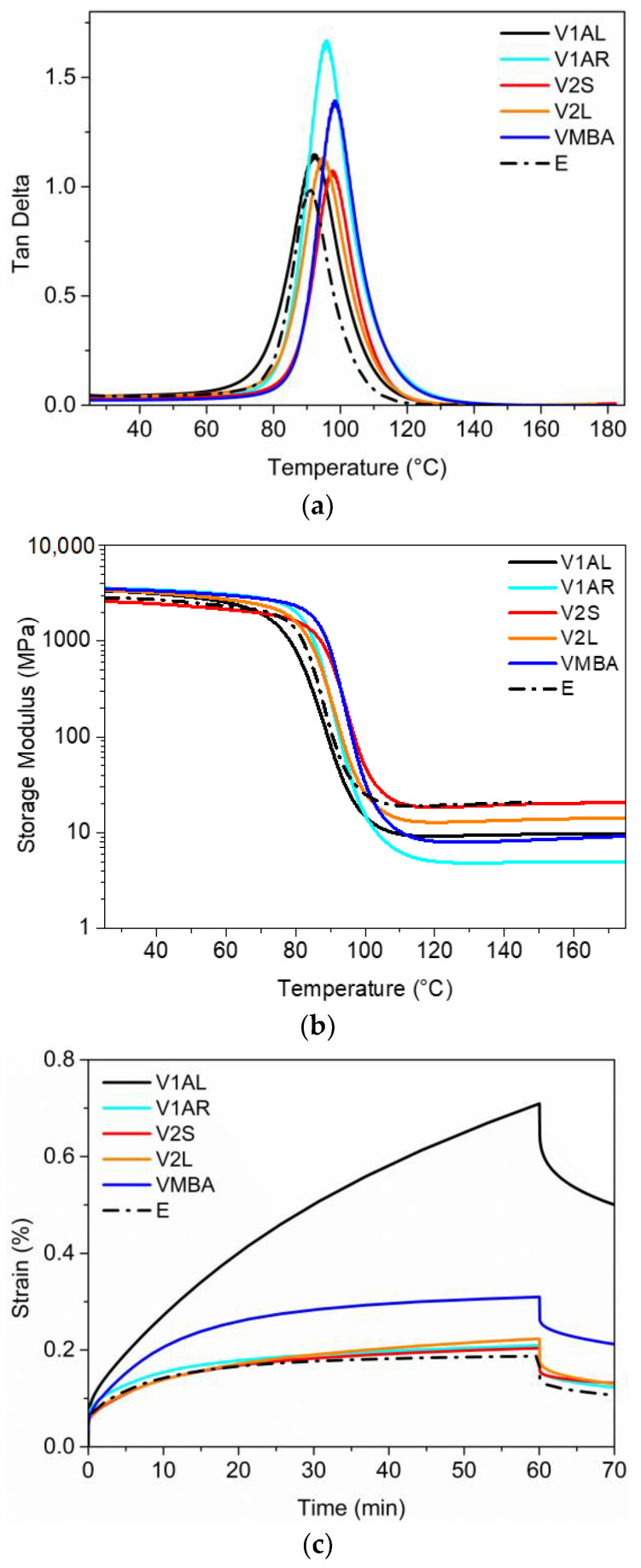
(**a**) DMA Tan δ, (**b**) storage modulus and (**c**) creep test of cured vitrimers tested at 60 °C under 1 MPa.

The viscoelastic properties of the cured vitrimers were analyzed via DMA. Temperature ramps were carried out to measure storage modulus (E′), loss modulus (E”) and the tan δ (E” to E′ ratio) of vitrimers (Figure 3a,b and Table 2). The peaks of tan δ of the vitrimers (Tg^DMA^) were positioned in the range of 92–98 °C. The half width of the tan δ peak (HWTDP) is related to the broadness of the relaxation time distribution. Narrow tan δ peaks indicate that all the mechanical relaxation occurs in a narrow temperature range and suggest all polymer segments of the network share a similar chemical environment. The measured thermal gap was between 13 and 17 °C for all materials. The height of tan δ gives an measure of the energy dissipation potential of the material. The highest peaks were observed for V1AR and VMBA, which displayed a more viscous character compared with the other prepared formulations. 

The distinctive feature of the designed vitrimers is the crosslink density (ν_C_) which describes the number of elastically effective network chains per unit volume of the sample. This could be calculated applying the rubber elasticity theory. However, for very highly crosslinked systems this approximation is not considered accurate [20]. The rubbery modulus is the key parameter that governs the value of ν_C_, i.e., the lower the modulus the lower the ν_C_. Thus, we take the value of the rubbery modulus as the indication of the crosslinking density. Among the vitrimers, the lowest rubbery modulus is shown by V1AR (5 MPa), whereas the largest is shown by V2S (19.5 MPa). Those results were in line with the expected network structure formed with the formulation components.

Creep experiments were also performed to compare the behavior of each vitrimer (Figure 3c). For the test 1 MPa stress was applied at 60 °C (bellow Tg) for 1 h. Among the five vitrimers, V1AL showed the highest strain deformation (0.71%) and did not reach a plateau, indicating that the material is relaxing under those conditions. It can be observed that at 60 °C the corresponding tan δ starts to rise, indicating that the network already has molecular segment mobility. The rest of the vitrimers and E reached a plateau and showed stability with the deformation: VMBA showed a strain of 0.31% and the rest of vitrimers remained below 0.2%. 

**Figure 4:**
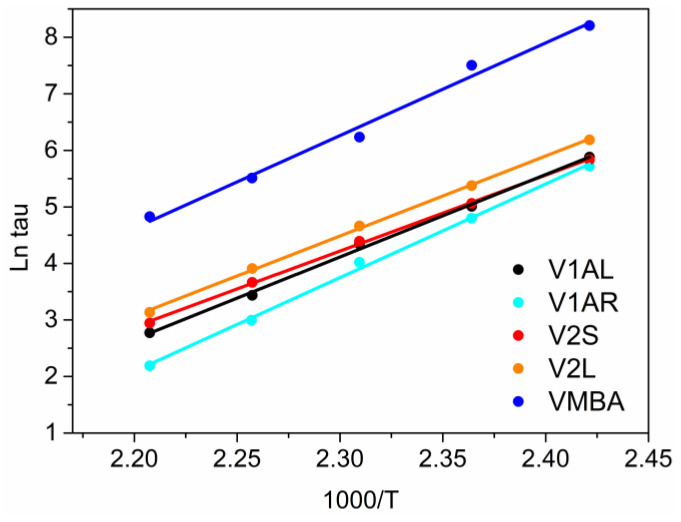
Fitting of the relaxation times to the Arrhenius’ equation.

The stress relaxation measurement of vitrimers is a key parameter to study the dynamic behavior of the network because it can be directly correlated with the molecular kinetics of the network (Table 4 and Figure 4). As it has been stated above, aromatic disulfide crosslinks undergo associative type exchange [16]. The stress relaxation of all prepared vitrimers was investigated in the DMA applying 1% tensile strain and monitoring the stress at different temperatures above Tg (140 °C, 150 °C, 160 °C, 170 °C and 180 °C) (Figures S4–S8). Relaxation time (τ*) was calculated following the Maxwell model which defines it as the time needed to decrease 67% of the starting storage modulus (E/E0 = 1/e). The recorded relaxation times, ln (τ), were plotted against 1000/T following the Arrhenius’s equation

τ(T) = τ0 · e^(Ea/RT)^

where τ is the relaxation time at each temperature, Ea is the activation energy, R is the gas constant (8.314 J mol^−1^ K^−1^) and T is the temperature. All plots present an Arrhenius linear correlation of ln (τ) with 1000/T in the measured temperature range. This means that the relaxation time is mainly governed by the temperature-dependent bond exchange mechanism.

As can be seen in Table 4, the analysis of the resulting relaxation times showed differences between all prepared vitrimers. A significant increase in relaxation time was obtained when part of the aromatic hardener was substituted with non-dynamic monoamine and thus, VMBA showed one order of magnitude slower relaxation compared to the other prepared vitrimers. This could be attributed to the low disulfide concentration in the network (see Table 2), and the difficulty to match and undergo associative exchange between two disulfide moieties. Additionally, in Table 4 the value of the residual stress is also given. The residual stress refers to the percentage of stress that was not relaxed after 1 h at 180 °C. Networks with 100% dynamic bonds should be capable of relaxing completely, thus the measured residual stress could be associated with the formation of non-reversible crosslinks during the polymerization reaction. The residual stress was remarkably high (8.5%) in the VMBA vitrimer which was cured with 4-AFD and a MBA hardener mixture. This could be attributed to some homopolymerization reactions that occurred when curing the network where a non-reversible network may be formed due to secondary reactions occurring.

Along with the Tg, the freezing topology temperature (Tv) is typically used to describe vitrimer systems. Those two temperatures need to be exceeded to trigger exchange reactions. Tv is a theoretically calculated temperature, and it is defined as the temperature below which the chemical exchange within the network is expected to be negligible; it is theoretically defined as the temperature at which the viscosity is 10^12^ Pa s [21]. Tv can be calculated using the Maxwell equation.

η = Gpτ*

where η is the viscosity (10^12^ Pa s), Gp is the plateau modulus (G = E′/2(1 + v); with v = 0.5) and τ* is the characteristic relaxation time. Three of the vitrimers showed Tv values below Tg^DMA^, whereas V1AL and VMBA showed slightly higher temperatures. We could conclude that in all networks the chemical exchange is protected primarily by the lack of the polymer segments’ mobility below Tg. 

The designed vitrimers allowed us to measure the effects of two network features on molecular exchange kinetics: (1) the effect of the crosslinking degree; and (2) the effect of disulfide concentration. Among vitrimers with the same Tg and disulfide concentration, networks with a lower crosslinking degree showed shorter relaxation times (V1AR < V1AL < V2L) and thus relaxed faster at low temperatures (140 °C). V1AR relaxes the fastest followed by V1AL and V2L. The exchange of disulfide is favored in this type of network with a lower viscosity and enhanced mobility due to the lower degree of covalent entanglements. These results are in good agreement with the results observed by Dichtel et al. [22] in polycarbonate vitrimers and Abu-Omar et al. for vitrimers with a transesterification bond exchange [23]. In transesterification-based vitrimers the opposite trend was observed. Recently, Hayashi et al. [17] demonstrated that when the concentrations of free OH groups and catalysts were kept the same among samples with different cross-link densities, lower relaxation times were obtained with increasing crosslink density. On the other hand, when bifunctional diluents were used and no-significant dangling groups were expected in the network, the faster relaxation was observed in V2S which is the vitrimer with the highest disulfide concentration (V2S < V2L < VMBA). These results showed that when no dangling groups are present in the network, in order to reduce the stress relaxation time, it is more important to increase the dynamic bonds in the network than lower the crosslink density. It was found that replacing part of the tetrafunctional amine hardener (4-AFD) with a bifunctional amine leads to long relaxation times, as well as to undesired side reactions, potentially homopolymerization reactions and the formation of covalent crosslinks (remaining residual stress). Leaving aside VMBA, the differences among the relaxation times of the rest of the vitrimers become less obvious at higher temperatures (180 °C) since the four vitrimers showed relaxation times below 20 s. 

### 3.3. Tensile and Flexural Properties

**Figure 5:**
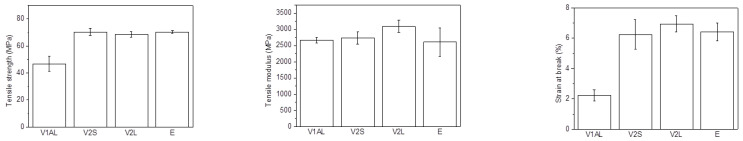
Tensile tests of epoxy matrices V1AL, V2S, V2L and E.

Tensile tests were performed at room temperature to characterize the mechanical properties of four vitrimers (V1AL, V1AR, V2S and V2L) and compared to the reference epoxy E (Table 5, Figure 5). As it can be seen, the addition of monofunctional or difunctional reactive diluents has different effects in the mechanical properties of prepared vitrimers. The addition of monofunctional reactive epoxy diluents led to a decrease in all mechanical properties [24]. V1AR showed high brittleness and the specimens broke out of standard, discarding the material from further analysis. Regarding V1AL vitrimer, the addition of a monofunctional long aliphatic epoxy diluent led to a decrease in both tensile strength and strain. It is well known that the addition of long aliphatic chains can led to a plasticization of the epoxy network, lowering the tensile strength. However, regarding strain at break, due to the plasticization effect a certain increase in strain at break can be expected. In this study a decrease in strain at break was obtained when adding both monofunctional reactive diluents. As it can be observed in other studies [25], the addition of monofunctional diluents led to an increase in strain at break up for 10% monofunctional reactive diluent. The addition of higher quantities, in contrast, can lead to a decrease in both stress and strain at break because it can create non-homogeneous zones decreasing the network integrity and creating free volumes in epoxy networks. On the contrary, when difunctional reactive diluents are used, no loss in mechanical properties was obtained and as can be seen in Table 5. V2S and V2L matched the tensile strength, modulus and strain at break of E. If we compare the crosslink density of networks prepared with monofunctional and difunctional reactive diluents and we compare them with the reference E epoxy resin, we can clearly observe that the crosslink density of the networks that contain bifunctional reactive diluents are similar to the reference E epoxy resin. The use of a monofunctional reactive diluent led to a decrease in crosslink density, enlarging the molecular weight between crosslinks. This change in the 3D network structure scan led to some distortions in the network lowering both the stress and strain at break. 

**Figure 6:**
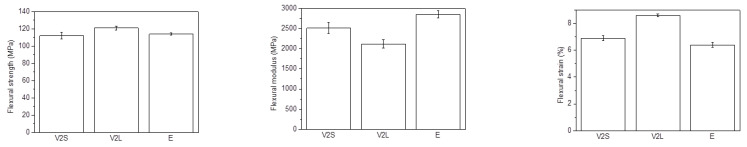
Flexural tests of epoxy matrices V2S, V2L and E.

The flexural properties of V2S, V2L and E were measured (Table 5, Figure 6). The vitrimers matched or exceed the flexural strength of E, showed a lower modulus and a higher flexural strain. 

The best-balanced overall performance was showed by vitrimers with bifunctional epoxy diluents (V2S and V2L). The main differences between them are the crosslinked density and the disulfide concentration, which are directly correlated. The disulfide concentration is 20% higher in V2S than in V2L. Although presenting relatively faster relaxation times in the low temperature range, at higher temperatures a similar relaxation could be achieved. 

## 4. Conclusions

In this paper five vitrimer networks showing comparable Tg (80–90 °C) were analyzed to determine in which the chemical structure extends, and how the crosslinking density and disulfide concentration affect the mechanical and dynamic properties of vitrimers showing comparable Tg. The thermal and mechanical properties were benchmarked with reference epoxy Araldite LY1564/Aradur 3486. The Tg-s were adjusted by reducing crosslink density and incorporating flexible segments in the networks as a straightforward strategy. From the mechanical performance perspective, the incorporation of monofunctional epoxy diluent weakened the tensile strength compared to the benchmark resin. In this regard, adding flexible bifunctional epoxy co-monomers to LY1564 resulted in a better option. When it comes to dynamic properties, two parameters, the crosslink density and disulfide molar concentration, were analyzed. Comparing systems with similar disulfide concentration, reducing the crosslinking density improves the relaxation presumably due to lower viscosity. While the increase in disulfide concentration led to faster relaxation times in the low temperature range, it could be compensated in higher temperature ranges. It was observed that low disulfide concentrations led to slow stress relaxation.

## Figures and Tables

**Table 1:** Chemical composition of conventional thermoset E and vitrimers.

Formulations	Weight Ratio					d_25 °C_ (10^6^ g·m^−3^)
	Reactive Diluent (g)		LY1564 (g)	Aradur (g)	4-AFD (g)	
E	-	-	100	34	-	1.137
V1AL	DY-E	20	80	-	40.01	1.182
V1AR	DY-K	30	70	-	39.58	1.202
V2S	DY-D	60	40	-	49.87	1.225
V2L	DGEPPG	40	60	-	38.43	1.224
VMBA	MBA	10.69	100	-	32.82	1.181

**Table 2:** Tan δ, HWTDT, E′ rubbery and creep.

Epoxy	Tan δ Max	HWTDP (°C)	E′ (MPa) at 140 °C	Strain (%) at 60 °C, 1 MPa	ν_C_ (mol·m^−3^)	M_C_ (g·mol^−1^)	[Disulfide] (mol·m^−3^)
E	0.98	14	20.3	0.18	1970	609	-
V1AL	1.13	17	9.5	0.71	922	1282	1362
V1AR	1.66	15	5.0	0.21	485	2478	1374
V2S	1.07	13	19.5	0.20	1893	647	1655
V2L	1.13	15	13.4	0.22	1300	941	1370
VMBA	1.38	15	8.14	0.31	790	1494	639

**Table 3:** Initial viscosities, curing enthalpy and thermal properties of cured epoxies.

Epoxy	Initial Viscosity (mPa.s) at:			Enthalpy (J/g)	Tpeak (°C)	Tg^DSC^ (°C)	Tg^DMA^ (°C)	Td^95 wt%^ (°C)	Water (%) Absorption
	25 (°C)	40 (°C)	60 (°C)						
E	598	80	23	530	82	86	91	286	3.3
V1AL	592	156	45	409	133	79	92	257	1.4
V1AR	657	166	48	463	136	87	96	282	2.1
V2S	238	88	32	523	132	89	98	266	4.3
V2L	997	256	71	429	132	83	95	246	2.7
VMBA	527	177	72	328	119	85	98	270	-

**Table 4:** Dynamic properties of vitrimers.

Epoxy	τ* (s) at:			Residual Stress (%)	Ea (KJ/mol)	Fitting Line (Arrhenius’)		Tv (°C)	Tg^DSC/DMA^ (°C)
	140 °C	160 °C	180 °C			Equation	R^2^		
V1AL	334	76	16	1.2	129	y = −29.42 + 15.58 x	0.99781	99	79/92
V1AR	306	56	9	0.0	137	y = −34.36 + 16.56 x	0.99711	74	87/96
V2S	342	81	19	1.2	111	y = −26.66 + 13.42 x	0.99893	75	89/98
V2L	486	106	23	1.3	117	y = −28.05 + 14.14 x	0.99882	77	83/95
VMBA	3666	510	125	8.5	136	y = −31.43 + 16.38 x	0.98605	98	85/98

**Table 5:** Tensile and flexural properties of epoxies.

Epoxy	Tensile			Flexural		
	Strength (MPa)	Modulus (MPa)	Strain at Break (%)	Strength (MPa)	Modulus (MPa)	Strain at Break (%)
E	70.3 σ 0.97	2610 σ 442	6.42 σ 0.60	114 σ 1.32	2850 σ 92	6.4 σ 0.18
V1AL	46.7 σ 5.6	2670 σ 86	2.23 σ 0.37	-	-	-
V2S	70.4 σ 2.6	2740 σ 192	6.25 σ 0.98	112 σ 3.80	2510 σ 138	6.9 σ 0.20
V2L	68.6 σ 2.04	3090 σ 191	6.95 σ 0.53	121 σ 2.30	2120 σ 101	8.6 σ 0.12

## Data Availability

Data will be made available on request.

## Supplementary Materials

The following supporting information can be downloaded at https://www.mdpi.com/article/10.3390/polym15204123/s1, Figure S1: FTIR spectra all prepared vitrimers; Figure S2: Curing thermograms of epoxy resins measured by DSC at 1 °C; Figure S3: Thermograms of cured vitrimers measured by TGA at 10 °C·min^−1^; Figure S4: Stress relaxation curves of V1AL measured by DMA at T (T = 140 °C, 150 °C, 160 °C, 170 °C, 180 °C) under 1% strain; Figure S5: Stress relaxation curves of V1AR measured by DMA at T (T = 140 °C, 150 °C, 160 °C, 170 °C, 180 °C) under 1% strain; Figure S6: Stress relaxation curves of V2S measured by DMA at T (T = 140 °C, 150 °C, 160 °C, 170 °C, 180 °C) under 1% strain; Figure S7: Stress relaxation curves of V2L measured by DMA at T (T = 140 °C, 150 °C, 160 °C, 170 °C, 180 °C) under 1% strain; Figure S8: Stress relaxation curves of VMBA measured by DMA at T (T = 140 °C, 150 °C, 160 °C, 170 °C, 180 °C) under 1% strain.
