# Supplementary material for: Analysis of the Effect of Network Structure and Disulfide Concentration on Vitrimer Properties
Source: Polymers (Basel). 2023 Oct 17;15(20):4123. doi: 10.3390/polym15204123 (PMC10610872; doi:10.3390/polym15204123)
Supplement: Supplementary file 1 [file polymers-15-04123-s001.zip › polymers-2553736-supplementary.pdf]

polymers

MDPI

Electronic supplementary information (ESI)

Analysis of the Effect of Network Structure and Disulfide Concentration on Vitrimer Properties

Itxaso Azcune [1], Edurne Elorza [1], Alaitz Ruiz de Luzuriaga [1], Arrate Huegun [1], Alaitz Rekondo [1,*] and Hans-Jürgen Grande [1,2]

[1] CIDETEC, Basque Research and Technology Alliance (BRTA), Paseo Miramón, 196, 20014 Donostia-San Sebastián, Spain; iazcune@cidetec.es (I.A.); eelorza@cidetec.es (E.E.); aruiz@cidetec.es (A.R.d.L.); ahuegun@cidetec.es (A.H.); hgrande@cidetec.es (H.J.G.)

[2] University of the Basque Country (UPV/EHU), Advanced Polymers and Materials—Physics, Chemistry and Technology Department, , Avda. Tolosa 72, 20018 Donostia-San Sebastian, Spain

* Correspondence: arekondo@cidetec.es

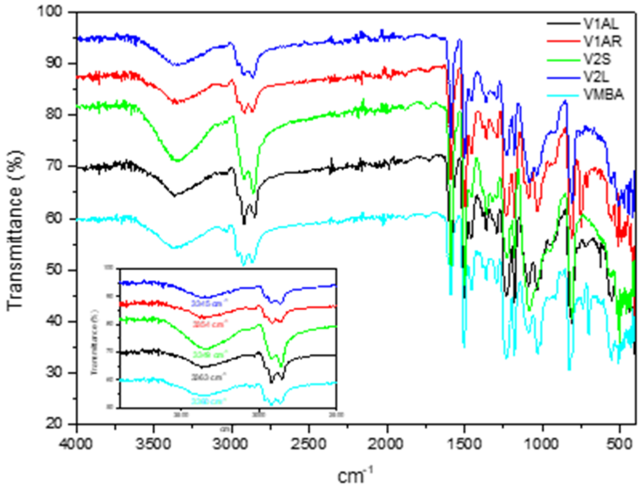

Figure S1: FTIR spectra all prepared vitrimers.

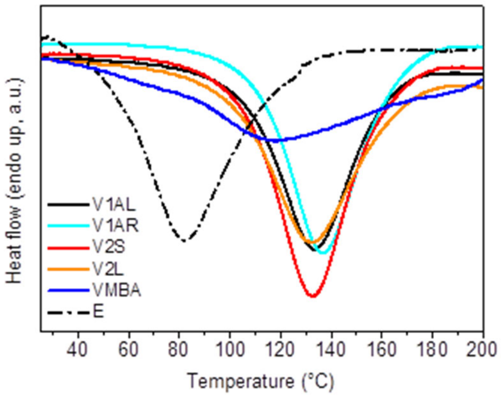

Figure S2: Curing thermograms of epoxy resins measured by DSC at 1 °C min^{-1} heating rate.

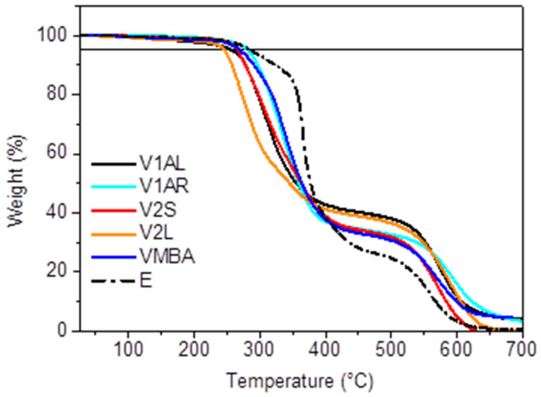

Figure S3: Thermograms of cured vitrimers measured by TGA at 10 °C min^{-1} heating rate under air atmosphere**.**

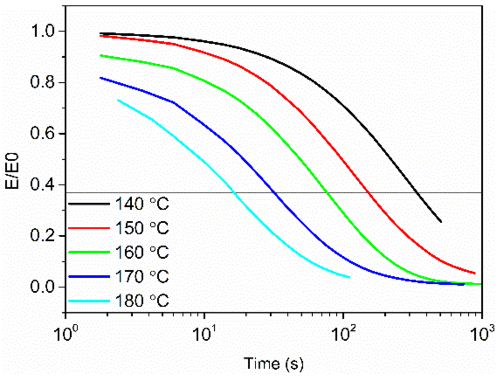

Figure S4: Stress relaxation curves of V1AL measured by DMA at T (T = 140 °C, 150 °C, 160 °C, 170 °C, 180 °C) under 1 % strain.

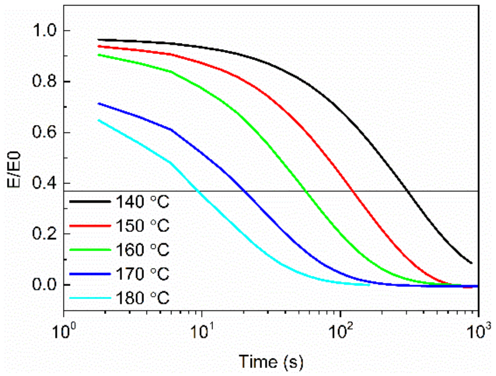

Figure S5: Stress relaxation curves of V1AR measured by DMA at T (T = 140 °C, 150 °C, 160 °C, 170 °C, 180 °C) under 1 % strain.

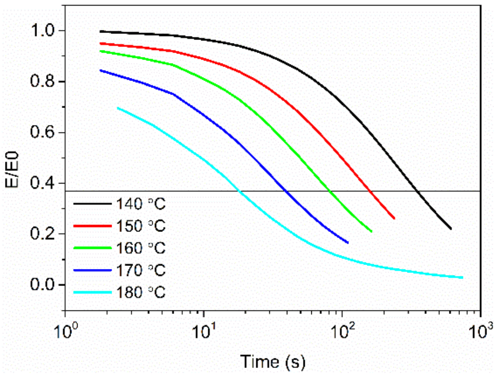

Figure S6: Stress relaxation curves of V2S measured by DMA at T (T = 140 °C, 150 °C, 160 °C, 170 °C, 180 °C) under 1 % strain.

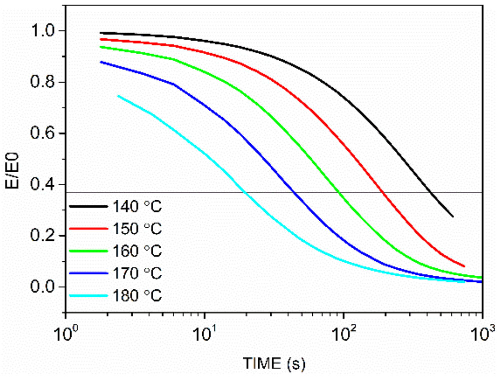

Figure S7: Stress relaxation curves of V2L measured by DMA at T (T = 140 °C, 150 °C, 160 °C, 170 °C, 180 °C) under 1 % strain.

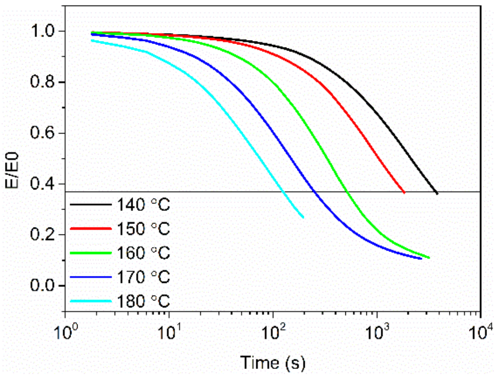

Figure S8: Stress relaxation curves of VMBA measured by DMA at T (T = 140 °C, 150 °C, 160 °C, 170 °C, 180 °C) under 1 % strain.
